# Supplementary material for: Red Dragon Fruit Peels: Effect of Two Species Ratio and Particle Size on Fibre Quality and Its Application in Reduced-Fat Alpaca-Based Sausages
Source: Foods. 2024 Jan 24;13(3):386. doi: 10.3390/foods13030386 (PMC10855916; doi:10.3390/foods13030386)
Supplement: Supplementary file 1 [file foods-13-00386-s001.zip › foods-2831448-supplementary.pdf]

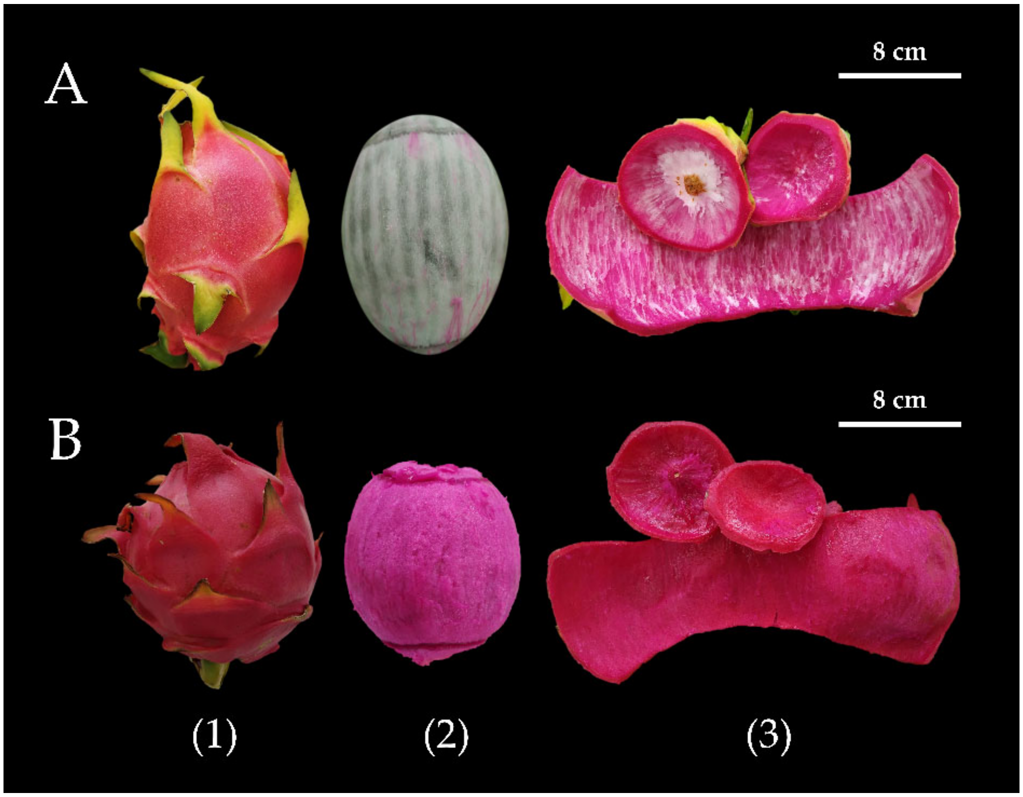

Figure S1. Red dragon fruit of (A) *Hylocereus undatus* and (B) *Hylocereus hybridum* species: Morphological features of the entire fruit (1), pulp (2), and peel (3).

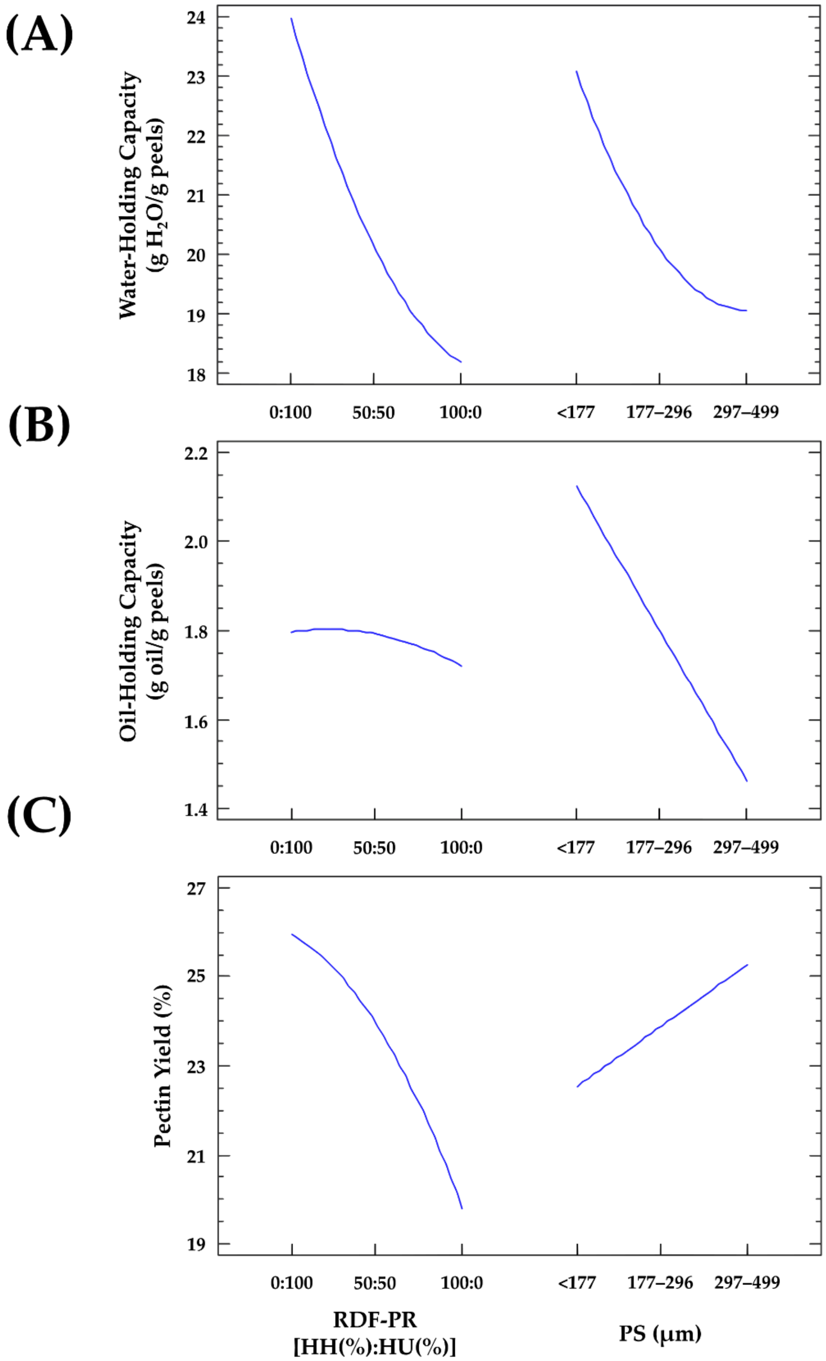

Figure S2. Plots of main effects of the optimised dependent variables: water-holding capacity (A), oil-holding capacity (B), and pectin yield (C).

Table S1. Characterisation of the physicochemical properties of red dragon fruit species (*Hylocereus undatus* and *Hylocereus hybridum*).

Physicochemical properties	Dragon fruit species	
	H. undatus	***H. hybridum***
LD (cm)	11.5 ± 0.13[a]	10.0 ± 0.25[b]
TD (cm)	7.79 ± 0.05[b]	8.46 ± 0.02[a]
W_{fruit} (g)	336 ± 9.65[b]	393 ± 10.59[a]
W_{peels} (g)	80.4 ± 1.01[a]	83.1 ± 2.89[a]
$W_{residues}$ (g)	11.6 ± 0.36[a]	6.31 ± 0.32[b]
W_{seeds} (g)	9.82 ± 0.66[a]	8.52 ± 0.19[b]
W_{pulp} (g)	235 ± 9.14[b]	290 ± 11.78[a]
Peel thickness (cm)	0.347 ± 0.01[b]	0.447 ± 0.03[a]
$Yield_{peels}$ (%)	23.9 ± 0.83[a]	22.1 ± 1.16[a]
$Yield_{residues}$ (%)	3.45 ± 0.04[a]	1.71 ± 0.13[b]
$Yield_{seeds}$ (%)	2.85 ± 0.30[a]	2.53 ± 0.24[a]
$Yield_{pulp}$ (%)	69.8 ± 0.72[a]	72.2 ± 2.07[a]
Moisture content (%)	88.2 ± 0.90[b]	91.1 ± 0.20[a]
TSS_{peels} (°Brix)	0.627 ± 0.01[a]	0.607 ± 0.006[a]
TSS_{pulp} (°Brix)	10.1 ± 0.12[a]	11.0 ± 0.72[a]
pH_{peels}	5.90 ± 0.06[a]	5.85 ± 0.04[a]
pH_{pulp}	4.60 ± 0.07[b]	4.84 ± 0.10[a]
Titratable acidity (%)	0.077 ± 0.004[b]	0.133 ± 0.004[a]

Data is expressed as means ± standard deviation (n=3). Different letters in the same row indicate significant differences (α=0.05) between red dragon fruit species. LD: Longitudinal diameter; TD: Transverse diameter; W: Weight; TSS: Total soluble solids. Titratable acidity is expressed in percentage of malic acid.

Table S2. 3^k full factorial design showing the independent variables: Red dragon fruit peels ratio (RDF-PR) and particle size (PS) at three levels.

Treatments	Coded values		Uncoded values	
	RDF-PR [HH:HU]	PS	RDF-PR [HH(%):HU(%)]	PS (μm)
T1	-1	-1	0:100	<177
T2	-1	0	0:100	177–296
T3	-1	+1	0:100	297–499
T4	0	-1	50:50	<177
T5	0	0	50:50	177–296
T6	0	+1	50:50	297–499
T7	+1	-1	100:0	<177
T8	+1	0	100:0	177–296
T9	+1	+1	100:0	297–499

HH (*Hylocereus hybridum*); HU (*Hylocereus undatus*).

Table S3. Pearson correlation coefficient (*r*) matrix of the colourimetric (L*, a*, b*, C, and h°), techno-functional (WHC, OHC, SC), and physicochemical (PY, CF, DE) properties of red dragon fruit peel powder (RDF-PP).

Dependent variables	L*	a*	b*	C	h°	WHC	OHC	SC	PY	CF	DE
**L***	1.0000	0.8331	-0.9120	0.8338	0.4293	0.5557	0.8709	0.9479	-0.1000	-0.6434	-0.6134
	p = —	p = < 0.0001	p = < 0.0001	p = < 0.0001	p = 0.0254	p = 0.0026	p = < 0.0001	p = < 0.0001	p = 0.6197	p = 0.0003	p = 0.0007
**a***	0.8331	1.0000	-0.8613	0.9999	0.4798	0.4267	0.9833	0.9234	-0.2706	-0.6501	-0.4065
	p = < 0.0001	p = —	p = < 0.0001	p = < 0.0001	p = 0.0113	p = 0.0264	p = < 0.0001	p = < 0.0001	p = 0.1723	p = 0.0002	p = 0.0353
**b***	-0.9120	-0.8613	1.0000	-0.8596	-0.3032	-0.3846	-0.8691	-0.9293	0.4005	0.7274	0.5817
	p = < 0.0001	p = < 0.0001	p = —	p = < 0.0001	p = 0.1242	p = 0.0476	p = < 0.0001	p = < 0.0001	p = 0.0384	p = < 0.0001	p = 0.0014
**C**	0.8338	0.9999	-0.8596	1.0000	0.4850	0.4249	0.9834	0.9228	-0.2679	-0.6511	-0.4070
	p = < 0.0001	p = < 0.0001	p = < 0.0001	p = —	p = 0.0103	p = 0.0272	p = < 0.0001	p = < 0.0001	p = 0.1767	p = 0.0002	p = 0.0351
**h°**	0.4293	0.4798	-0.3032	0.4850	1.0000	0.1305	0.4917	0.4324	0.1237	-0.5711	-0.2749
	p = 0.0254	p = 0.0113	p = 0.1242	p = 0.0103	p = —	p = 0.5166	p = 0.0092	p = 0.0243	p = 0.5388	p = 0.0019	p = 0.1652
**WHC**	0.5557	0.4267	-0.3846	0.4249	0.1305	1.0000	0.5059	0.6058	0.6022	0.1150	-0.2728
	p = 0.0026	p = 0.0264	p = 0.0476	p = 0.0272	p = 0.5166	p = —	p = 0.0071	p = 0.0008	p = 0.0009	p = 0.5678	p = 0.1686
**OHC**	0.8709	0.9833	-0.8691	0.9834	0.4917	0.5059	1.0000	0.9404	-0.1744	-0.6411	-0.3958
	p = < 0.0001	p = < 0.0001	p = < 0.0001	p = < 0.0001	p = 0.0092	p = 0.0071	p = —	p = < 0.0001	p = 0.3844	p = 0.0003	p = 0.0409
**SC**	0.9479	0.9234	-0.9293	0.9228	0.4324	0.6058	0.9404	1.0000	-0.1216	-0.6273	-0.5591
	p = < 0.0001	p = < 0.0001	p = < 0.0001	p = < 0.0001	p = 0.0243	p = 0.0008	p = < 0.0001	p = —	p = 0.5456	p = 0.0005	p = 0.0024
**PY**	-0.1000	-0.2706	0.4005	-0.2679	0.1237	0.6022	-0.1744	-0.1216	1.0000	0.5426	0.1436
	p = 0.6197	p = 0.1723	p = 0.0384	p = 0.1767	p = 0.5388	p = 0.0009	p = 0.3844	p = 0.5456	p = —	p = 0.0035	p = 0.4748
**CF**	-0.6434	-0.6501	0.7274	-0.6511	-0.5711	0.1150	-0.6411	-0.6273	0.5426	1.0000	0.5577
	p = 0.0003	p = 0.0002	p = < 0.0001	p = 0.0002	p = 0.0019	p = 0.5678	p = 0.0003	p = 0.0005	p = 0.0035	p = —	p = 0.0025
**DE**	-0.6134	-0.4065	0.5817	-0.4070	-0.2749	-0.2728	-0.3958	-0.5591	0.1436	0.5577	1.0000
	p = 0.0007	p = 0.0353	p = 0.0014	p = 0.0351	p = 0.1652	p = 0.1686	p = 0.0409	p = 0.0024	p = 0.4748	p = 0.0025	p = —

WHC: water-holding capacity (g/g peels); OHC: oil-holding capacity (g oil/g peels); SC: swelling capacity (ml H_2O/g peels); PY: pectin yield (%); CF: crude fibre (%); DE: degree of esterification (%). "–": indicates a lack of statistically significant association between the same dependent variables.

Table S4. Sensory properties of emulsified alpaca-based sausages with different substitution levels of pork-back fat by the optimal red dragon fruit peel powder (RDF-PP) from treatment one (T1).

Treatment	Appearance	Colour	Odour	Flavour	Texture	Overall acceptability
**C**	5.51 ± 1.50 ^{a}	5.44 ± 1.48 ^{a}	5.48 ± 1.85 ^{a}	5.07 ± 1.76 ^{b}	5.43 ± 1.73 ^{b}	5.51 ± 1.69 ^{b}
**OF3**	5.85 ± 1.68 ^{a}	5.83 ± 1.68 ^{a}	5.93 ± 1.90 ^{a}	5.89 ± 1.76 ^{a}	6.12 ± 1.64 ^{a}	6.27 ± 1.63 ^{a}

Mean values with different superscript letters within a column indicate significant difference (α=0.05) between treatments. C: control formulation of emulsified alpaca-based sausages with 0.00% hydrated RDF-PP; OF3: optimal formulation 3 (with 9.86% RDF-PP).

Table S5. Mann-Whitney U test for the sensory properties of emulsified alpaca-based sausages with different pork-back fat substitution levels between: control formulation of emulsified alpaca-based sausages with 0.00% hydrated RDF-PP (C) and optimal formulation 3 (OF3).

Variable	Rank Sum C	Rank Sum OF3	U	Z	Z *p*-value	Z adj.	Z adj. *p*-value	Valid N C	Valid N OF3
**Appearance**	5249.0	6076.0	2399.0	-1.55	0.1205	-1.58	0.1143	75	75
**Colour**	5287.0	6038.0	2437.0	-1.41	0.1586	-1.44	0.1512	75	75
**Odour**	5231.0	6094.0	2381.0	-1.62	0.1052	-1.64	0.1009	75	75
**Flavour**	4911.5	6413.5	2061.5	-2.82	0.0047	-2.86	0.0042	75	75
**Texture**	5025.0	6300.0	2175.0	-2.39	0.0166	-2.43	0.0151	75	75
**Overall acceptability**	4945.5	6379.5	2095.5	-2.69	0.0070	-2.73	0.0063	75	75

U: Mann-Whitney U statistic; Z: Z-score; Z adj.: Z adjusted; N: total number of experiments; C: control formulation of emulsified alpaca-based sausages with 0.00% hydrated RDF-PP; OF3: optimal formulation 3 (with 9.86% RDF-PP).

Table S6. Pork-back fat (%), red dragon fruit peel powder (RDF-PP), and water content of emulsified alpaca-based sausages based on a 2000 g formulation.

Formulation	Pork-back fat (%)	RDF-PP (g)	RDF-PP (%)	Water (g)	Water (%)
C	15.0	0.00	0.00	0.00	0.00
F1	11.7	2.00	0.10	63.7	3.19
F2	8.43	4.00	0.20	127.4	6.37
**F3**	**5.14**	**6.00**	**0.30**	**191.2**	**9.56**

C: control formulation of emulsified alpaca-based sausages with 0.00% hydrated RDF-PP; F1: emulsified alpaca-based sausages with 3.29% hydrated RDF-PP; F2: emulsified alpaca-based sausages with 6.57% hydrated RDF-PP; F3: emulsified alpaca-based sausages with 9.86% hydrated RDF-PP.
